# The Aβ protofibril selective antibody mAb158 prevents accumulation of Aβ in astrocytes and rescues neurons from Aβ-induced cell death

**DOI:** 10.1186/s12974-018-1134-4

**Published:** 2018-03-28

**Authors:** Sofia Söllvander, Elisabeth Nikitidou, Linn Gallasch, Marlena Zyśk, Linda Söderberg, Dag Sehlin, Lars Lannfelt, Anna Erlandsson

**Affiliations:** 1 Department of Public Health and Caring Sciences, Molecular Geriatrics, Rudbeck Laboratory, Uppsala University, Uppsala, Sweden; 2 BioArctic AB, Warfvinges väg 35, SE-112 51 Stockholm, Sweden

**Keywords:** Alzheimer’s disease, Amyloid-β, Antibody, Clearance, Astrocyte, Neuron

## Abstract

**Background:**

Currently, several amyloid beta (Aβ) antibodies, including the protofibril selective antibody BAN2401, are in clinical trials. The murine version of BAN2401, mAb158, has previously been shown to lower the levels of pathogenic Aβ and prevent Aβ deposition in animal models of Alzheimer’s disease (AD). However, the cellular mechanisms of the antibody’s action remain unknown. We have recently shown that astrocytes effectively engulf Aβ_42_ protofibrils, but store rather than degrade the ingested Aβ aggregates. In a co-culture set-up, the incomplete degradation of Aβ_42_ protofibrils by astrocytes results in increased neuronal cell death, due to the release of extracellular vesicles, containing N-truncated, neurotoxic Aβ.

**Methods:**

The aim of the present study was to investigate if the accumulation of Aβ in astrocytes can be affected by the Aβ protofibril selective antibody mAb158. Co-cultures of astrocytes, neurons, and oligodendrocytes, derived from embryonic mouse cortex, were exposed to Aβ_42_ protofibrils in the presence or absence of mAb158.

**Results:**

Our results demonstrate that the presence of mAb158 almost abolished Aβ accumulation in astrocytes. Consequently, mAb158 treatment rescued neurons from Aβ-induced cell death.

**Conclusion:**

Based on these findings, we conclude that astrocytes may play a central mechanistic role in anti-Aβ immunotherapy.

**Electronic supplementary material:**

The online version of this article (10.1186/s12974-018-1134-4) contains supplementary material, which is available to authorized users.

## Background

Alzheimer’s disease (AD) is a devastating neurodegenerative disease, affecting millions of people worldwide. Clinically, AD is characterized by the progressive loss of neurons, which typically leads to severe impairments in cognitive functions, including learning and memory. Key pathological features of AD include the formation of amyloid beta (Aβ) plaques, neurofibrillary tangles, neuronal loss, and reactive gliosis.

Due to its hydrophobic nature, the Aβ peptide has a strong propensity to aggregate and form both soluble Aβ aggregates and insoluble Aβ fibrils, which eventually deposit as plaques. Results from many research groups indicate that the widespread neuronal dysfunction in the AD brain is caused by soluble Aβ oligomers/protofibrils, rather than the insoluble Aβ fibrils [1–3]. How the soluble Aβ aggregates exert their toxic effects is however not clear, but they have been shown to inhibit long-term potentiation and to impair synaptic function and plasticity [1, 4–10]. Moreover, results from biophysical studies indicate that Aβ oligomers may perform their toxic function through interactions with the lipid bilayers, since membrane interaction can promote formation of toxic Aβ species [11–13].

Immunotherapy has emerged as a promising method to reduce Aβ pathology. Due to their toxic nature, soluble Aβ aggregates have been suggested to be an especially attractive immunization target. Currently, several anti-Aβ antibodies, including the Aβ protofibril selective monoclonal antibody BAN2401, are evaluated as AD therapeutics [14]. Importantly, prefibrillar forms of Aβ have been shown to be the predominant species of soluble Aβ aggregates in both transgenic mice and human AD brains [15]. In animal models of AD, the murine version of BAN2401, mAb158, lowers brain levels of soluble Aβ protofibrils and prevents Aβ deposition [16], but the underlying mechanisms and the role of different cell types for its action remain to be elucidated.

There is compelling evidence that Aβ pathology is closely associated with inflammation, and reactive astrocytes and microglia are situated tightly around the plaques [17]. Being the most abundant glial cell type in the nervous system, astrocytes play an important role in maintaining brain homeostasis [18]. Their functions include metabolic support of neurons, modification of synapse signaling, recycling of neurotransmitters, blood–brain barrier regulation, and glymphatic clearance [18–20]. In addition, astrocytes respond to neurodegenerative disorders, including AD, through astrogliosis, a process in which they convert to a reactive inflammatory state [21, 22]. The complex role of astrocytes in the pathological brain is largely depending on their release and uptake of substances from the microenvironment that they share with the neurons [18]. For example, astrocytes confer neuroprotection by removing excessive extracellular glutamate, potassium, and calcium, while they produce cytokines and chemokines that could be harmful to neurons, if chronically released [18, 23].

Reactive astrocytes effectively engulf dead cells and damaged synapses and protein aggregates [24–31]. Interestingly, astrocytes have been shown to be more efficient than microglia in taking up Aβ, particularly during the early stages of AD [32]. The fact that reactive astrocytes with high Aβ load are frequently found in the human AD brain, further confirms the importance of astrocytes in Aβ clearance [33]. Yet, the therapeutic potential of astrocytes remains to be investigated. In a recent study, we demonstrated that astrocytes engulf large amounts of Aβ_42_ protofibrils that are accumulated rather than digested by the cells. This intracellular storage of Aβ results in severe astrocytic endosomal/lysosomal defects and secretion of extracellular vesicles with neurotoxic content [31].

The aim of the present study was to investigate if degradation of Aβ_42_ protofibrils by astrocytes can be enhanced by treatment with the Aβ protofibril selective antibody mAb158. Our results demonstrate that the antibody substantially increases clearance of pathological Aβ by astrocytes and rescues neurons from Aβ-induced cell death. This is important data, highlighting a possible role for astrocytes in the cellular response to Aβ immunotherapy.

## Methods

### Synthetic Aβ_42_ protofibrils

Synthetic Aβ_42_ protofibrils used in this study were prepared according to a well-established protocol [15, 31, 34, 35]. Synthetic Aβ_42_ peptides (American Peptide Company Inc.) dissolved in 10 mM NaOH were neutralized with 10× phosphate-buffered saline (PBS) to 443 μM (2 mg/ml) and incubated 30 min at 37 °C. Fluorescent HiLyte™ Fluor 555-labeled Aβ_42_ (Aβ_42_-555) peptides (Anaspec Inc) were diluted in 10×PBS to a concentration of 36 μM, followed by incubation for 4 h at 37 °C. Both unlabeled Aβ_42_ protofibrils and Aβ_42_-555 protofibrils were centrifuged for 5 min at 17900 × *g* to remove any insoluble aggregates. The purity (> 95%) of the Aβ_42_ protofibril preparation was analyzed by HPLC-SEC, using a Superdex 75 column. The SEC chromatogram for the specific batch of protofibrils used in this study is included in our previous paper [31].

### Animals

All experiments involving animals were performed at Uppsala University, Sweden. The experiments were approved by the Uppsala County Animal Ethics Board (ethical permit number: C75/13, valid 2013-06-28 to 2018-06-28), following the rules and regulations of the Swedish Animal Welfare Agency, in compliance with the European Communities Council Directive of 22 September 2010 (2010/63/EU). The mice were housed at the animal facility at Uppsala University Hospital, Uppsala, in a 12–12 dark–light cycle. The mice were kept in an enriched environment and given water and food ad libitum.

### Co-cultures of neurons and glia

Cerebral cortices from C57/BL6 mice of embryonal day 14 (E14) were dissected in Hank’s buffered salt solution supplemented with 50 U/ml penicillin, 50 mg/ml streptomycin, and 8 mM Hepes buffer (HBSS, all from Invitrogen). The cortices were dissociated in fresh HBSS, centrifuged at 150 × *g* and resuspended in cell culture medium. The cells were expanded in DMEM/F12-GlutaMAX supplemented with 1× B27 supplement, 50 U/ml penicillin, 50 mg/ml streptomycin, and 8 mM Hepes buffer, 10 ng/ml bFGF (all from ThermoFisher) and 20 mg/ml EGF (VWR). Neurospheres were passaged every second or third day by dissociation in HBSS and resuspended in medium with bFGF and EGF. Prior to experiments, the cells were plated as a monolayer, at a concentration of 1.5 × 10^5^ cells/ml, on coverslips (In Vitro Diagnostics) or cell culture dishes (Falcon), coated with Poly-L-Ornithine (Sigma-Aldrich) and Laminin (Invitrogen). After 24 h, the medium was replaced with mitogen-free medium to initiate cell differentiation to a mixed population of neurons, astrocytes, and oligodendrocytes. Neural stem cells have the capacity to differentiate to neurons, astrocytes, and oligodendrocytes, but not microglia [36–38]. To verify that no microglia were present in the cultures, we performed immunocytochemistry with a specific antibody to Iba-1 (Additional file 1). A brain tissue section from a 16-month-old APP_ArcSwe_ mouse was included as a positive control. During the 7-day differentiation period, the cell culture medium was changed every second or third day. Only neurospheres from passage 2–4 were used for experiments. Cells were kept at 37 °C in 5% CO_2_ atmosphere.

### Antibody fragmentation

Recombinant mAb158 (RmAb158) was cleaved by the FragIT kit (Genovis AB) according to manufacturer’s guidelines to generate F(ab’)_2_–RmAb158, as previously described [39]. The kit produces a homogenous preparation of F(ab’)_2_ fragments by using the bacterial enzyme FabRICATOR (IdeS), cleaving IgG at a specific site just below the hinge region. Fc fragments and non-cleaved antibody were removed from the F(ab’)_2_ fragments with a CaptureSelect Fc affinity resin (ThermoFisher). The products were analyzed by SDS-PAGE under non-reducing conditions to confirm appropriate cleavage (size) and purity of the fragments. Briefly, samples were mixed with Laemmli buffer, loaded onto a NuPage Bis-Tris 4–12% gel (ThermoFisher) and run at 200 V for 22 min. The gel was stained with Page Blue (Fermentas) for 1 h followed by extensive washes in milli-Q water.

### Aβ stimulation and antibody treatment

Co-cultures of neurons and glia were exposed to 0.1 μM Aβ_42_ protofibrils (either 555-labeled or unlabeled) for 24 h. Controls received fresh cell culture medium without Aβ_42_ protofibrils. For the antibody treatment, 0.1 μM Aβ_42_ protofibrils were incubated for 5 min with 13 nM of the hybridoma-produced murine mAb158 (IgG_2a_) or the recombinantly produced mAb158, RmAb158 (IgG_2c_), with or without the N297D mutation (all from BioArctic AB), before being added to the cultures. As negative control antibodies, the irrelevant antibody Ly128 (IgG_1_, Mabtech), recognizing flagellin in bacteria, and MOPC-173 (IgG_2a_, BD Pharmingen), with unknown specificity, were used. It has been proven that Ly-128 does not cross-react with Aβ [16]. For analysis of the Aβ_42_ concentration in media, a lower concentration (0.01 μM Aβ_42_ protofibrils) and reduced volume (400 μl) of the cell culture media were added to the co-cultures. The Aβ_42_ protofibrils/Aβ_42_-555 protofibrils and the various antibodies were added to the co-cultures in a volume of 2 ml per well, except when analyzing medium concentrations of Aβ (then 400 μl were used). Following 24-h treatment, the co-cultures were washed in cell culture media three times and the cell-containing coverslips were transferred to new culture dishes. The cells were fixed (24 h), lysed (24 h), or cultured for additional 6 (24 h + 6 days) or 12 days (24 h + 12 days) in Aβ-free cell culture medium prior to fixation or cell lysis. To analyze the effect of mAb158 on Aβ_42_ protofibril degradation further, mAb158 was added 3 days after the 24 h Aβ_42_-555 protofibril exposure before cultured for additional 6 (24 h + 6 days) or 12 days (24 h + 12 days) and washed thoroughly in Aβ-free cell culture medium prior to fixation. Further, in another control experiment, co-cultures were incubated with mAb158 for 1 h and then extensively washed in cell culture media, prior to 24 h Aβ_42_ protofibril exposure.

### Lysosomal and proteosomal inhibition

In order to investigate the influence of the proteosomal and endosomal–lysosomal pathway on mAb158-mediated Aβ reduction in astrocytes, we preincubated co-cultures with the proteosomal inhibitor mg-132 (Calbiochem, Millipore, 10 μM) or the lysosomal inhibitor Bafilomycin (Calbiochem, Millipore, 200 nM) for 30 min prior to Aβ_42_ protofibril exposure ± mAb158 treatment. The inhibitors remained in the media during the 24-h exposure. Following fixation, the accumulation of Aβ was assessed by immuno-fluorescence. The inhibitors did not have any apparent toxic effects in the concentration and exposure times used.

### Immunostaining of cell cultures

Coverslips were fixed for 15 min in RT with 4% paraformaldehyde and permeabilized and blocked with 0.1% Triton X-100 (both from Sigma-Aldrich) and 5% normal goat serum (NGS, Bionordika) in PBS for 30 min at RT. Primary antibodies were incubated in 0.1% Triton X-100 with 0.5% NGS for 1–4 h at RT or O/N at 4 °C. Thereafter, the coverslips were thoroughly washed in PBS three times between each step. Incubation with secondary antibodies was performed in 0.1% Triton X-100 and 0.5% NGS for 45 min at 37 °C. The following primary antibodies were used in the study: rabbit anti-glial fibrillary acidic protein (GFAP, 1:400, DakoCytomation), mouse anti-GFAP (1:400, Sigma-Aldrich), mouse anti-βIII-tubulin (1:200, Covance), rabbit anti-Iba-1 (1:200, Abcam), polyclonal rabbit anti-Aβ_42_ (1:200, Invitrogen), and monoclonal mouse anti-Aβ antibody 6E10 (10 μg/ml, epitope: 3–8, Covance). Secondary antibodies used were AlexaFluor 488 and 555, all against mouse or rabbit (1:200, Molecular probes), and AlexaFlour 488, anti-IgG_2a_ antibody against mouse (1:200, Life Technologies). Phalloidin-Fluorescein (2 μM, Sigma-Aldrich) was used for actin visualization. The coverslips were mounted on microscope glass slides using Vectashield hard-set mounting medium with 4′,6-diamidino-2-phenylindole (DAPI, DAKO). A Zeiss Observer Z1 Microscope and Carl Zeiss LSM700 confocal microscope (Zeiss) were used for analysis. Images and confocal z-stacks were visualized with Zen 2012 software.

### Immunostaining of tissue sections

The analysis of Iba-1 included immunohistochemistry of tissue sections from a 16-month-old APP_ArcSwe_ mouse, as a positive control. In short, cryostat brain sections, prepared as previously described [40], were rehydrated in PBS and permeabilized and blocked with 0.3% Triton X-100 (Sigma-Aldrich) and 5% NGS (Bionordika) in PBS for 1 h at RT. The sections were incubated with the primary antibody (rabbit anti-Iba-1, 1:200, Abcam) in 0.5% NGS in PBS O/N at 4 °C. Sections were washed three times for 5 min in PBS. Incubation with secondary antibody (anti-rabbit Alexa Fluor 488, 1:200, Invitrogen) was performed in 0.5% NGS in PBS for 1 h at 37 °C, followed by three washes for 5 min in PBS. Sections were mounted onto microscope glass slides (Menzel Gläser) using EverBrite mounting medium containing DAPI (Biotium).

### Time-lapse experiments

Time-lapse experiments were performed at 37 °C in humidified 5% CO_2_ atmosphere, using a Nikon Biostation IM Live Cell Recorder (Nikon). The cells were cultured at a concentration of 1.5 × 10^5^ cells/ml, in time-lapse culture dishes (VWR), and pictures were taken every 10th minute for up to 24 h. Aβ_42_-555 protofibrils and mAb158 labeled with DyLight™ 488 (ThermoFisher) were used in the time-lapse experiments.

### Cell medium and lysates

For Aβ quantification studies, neurons and glia were cultured at a concentration of 2.4 × 10^5^ cells/ml in cell culture dishes (Corning). Following collection of the cell culture medium, the co-cultures were lysed by addition of ice-cold lysis buffer (20 mM Tris pH 7.5, 0.5% Triton X-100, 0.5% deoxycholic acid, 150 mM NaCl, 10 mM EDTA, 30 mM NaPyroP and protease inhibitor (Roche)) to the dish. The lysed cells were collected using a cell lifter (Corning Inc.), transferred to Eppendorf tubes, incubated on ice for 30 min, and centrifuged (30 min, 4 °C, 12000 × *g*). The medium and cell lysates were stored at − 70 °C until the time of analysis by ELISA or Western blot.

### Aβ_1-x_ and Aβ_x-42_ ELISAs

For Aβ_1-x_ ELISA, 96 well EIA/RIA plates (Corning Inc.) were coated O/N at 4 °C with the N-terminus specific (epitope 1–5) antibody mAb82E1 (100 ng/well, IBL-Hamburg) in PBS. Plates were blocked with 1% bovine serum albumin (BSA) in PBS for 2 h at RT. Standard series of synthetic Aβ_42_ monomers (American Peptide) and samples were denatured by boiling for 5 min in 0.5% sodium dodecyl sulfate (SDS) to avoid impaired detection caused by aggregated Aβ [41]. All SDS-treated samples were diluted x10 to decrease the SDS concentration and avoid SDS interference in the ELISAs. Washing was performed by adding 250 μl washing buffer (phosphate-buffered NaCl with 0.1% Tween-20 and 0.15% Kathon) times three repetitions between each step of the ELISA. All dilutions occurred in ELISA incubation buffer (0.05% Tween, 0.1% BSA, and 0.15% Kathon in PBS at pH 7.4). Plates were incubated for 2 h before adding biotinylated 4G8 (0.3 μg/ml, Covance), specific to the mid-region of Aβ, as secondary antibody for 1 h. Thereafter, plates were incubated with streptavidin coupled HRP (1:2000, Mabtech AB) for 1 h. K-blue enhanced (Neogen Corporation) was used as HRP substrate, and the reaction was stopped with 1 M H_2_SO_4_. Plates were measured by Tecan Infinite M200 PRO spectrophotometer (Tecan Group Ltd.) at 450 nm and analyzed with Magellan v7.0 software (Tecan Group Ltd.). For the Aβ_x-42_ ELISA, polyclonal Aβ_42_ antibody (100 ng/well, Agrisera) and biotinylated 4G8 (0.5 μg/ml) were used as primary and secondary antibody, respectively. Aβ_x-42_ ELISA was performed according to the same protocol as the Aβ_1-x_ ELISA, except for prolonged incubation times for blocking, sample (O/N, 4 °C) and secondary antibody (2 h, RT) and increased SA-HRP dilution (1:5000).

### Western blot analysis

Medium from the co-cultures was mixed with NuPage sample buffer and NuPage reducing agent (both from ThermoFisher) and incubated for 5 min at 95 °C. Chameleon duo marker (Li-Cor) was used as a ladder, and medium was loaded according to maximal volume (30 μl/well) on a NuPage Bis-Tris 4–12% gel (ThermoFisher) and run at 200 V for 30 min in MES SDS running buffer (ThermoFisher), followed by transfer to nitrocellulose membrane (Bio-Rad) at 25 V for 7 min using a Trans-Blot Turbo system (Bio-Rad). The membrane was briefly washed in Tris-buffered saline (TBS) and blocked in 5% nonfat dry milk in 0.1% TBS-Tween for 1 h at RT, before the primary antibody (polyclonal rabbit anti-Aβ_42_, 1:2000, Invitrogen) was added and incubated O/N at RT on shaking. The membrane was washed in 0.1% TBS-Tween for 2 × 5 min and 2 × 10 min prior to 1 h incubation with secondary anti-rabbit antibody conjugated with horseradish peroxidase (Pierce) in 0.1% TBS-Tween. The enhanced chemiluminescence (ECL) system (SuperSignal West Dura Extended Duration Substrate, ThermoFisher) was used for development and imaging, and analysis of bands was performed using an ImageQuant 400 GE Odyssey (Li-Cor). The membrane was re-probed with secondary anti-mouse antibody. Briefly, the membrane was blocked in Odyssey blocking buffer (TBS) (Li-Cor) for 1 h at RT and incubated with DyLight 800 secondary anti-mouse antibody (1:20000, Invitrogen) in Odyssey blocking buffer (TBS) and 0.1% TBS-Tween (1:1) for 1 h at RT. Three washes in 0.1% TBS-Tween and one wash in TBS followed, before the signal was detected using the Odyssey Sa Imaging system (Li-Cor).

### Area and intensity measures of Aβ inclusions and cell counting

For intensity and area measurements of Aβ-555, 30 images (ten images/coverslip from three independent cell cultures) were captured with an *×* 40 objective on a Zeiss Observer Z1, using the same settings. The images were analyzed with the Zen 2012 software (Zeiss), and all area and intensity measurements were set manually. For analysis of neuronal survival, 30 images (ten images/coverslip from three independent cell cultures) were captured with an *×* 20 objective on a Zeiss Observer Z1. The number of viable, βIII-tubulin positive neurons was manually quantified in each field. All images were analyzed in a blinded fashion.

### Statistics

All experiments were performed in triplicates with independent co-cultures derived from embryos of different pregnant mice. The results are presented in scatter plots or box plots with mean ± standard deviation. Since the data were found not to meet the assumption of normal distribution using the Shapiro–Wilk’s *W* test, Kruskal–Wallis ANOVA was used and was followed by Mann–Whitney *U* test for pairwise comparisons. Western blot intensity measurements were statistically analyzed using one-way ANOVA followed by Tukey’s multiple comparison test. Level of significance were set to *P < 0.05, **P < 0.01, and ***P < 0.001.

## Results

### The Aβ protofibril selective antibody mAb158 prevents Aβ accumulation in astrocytes

**Fig. 1:**
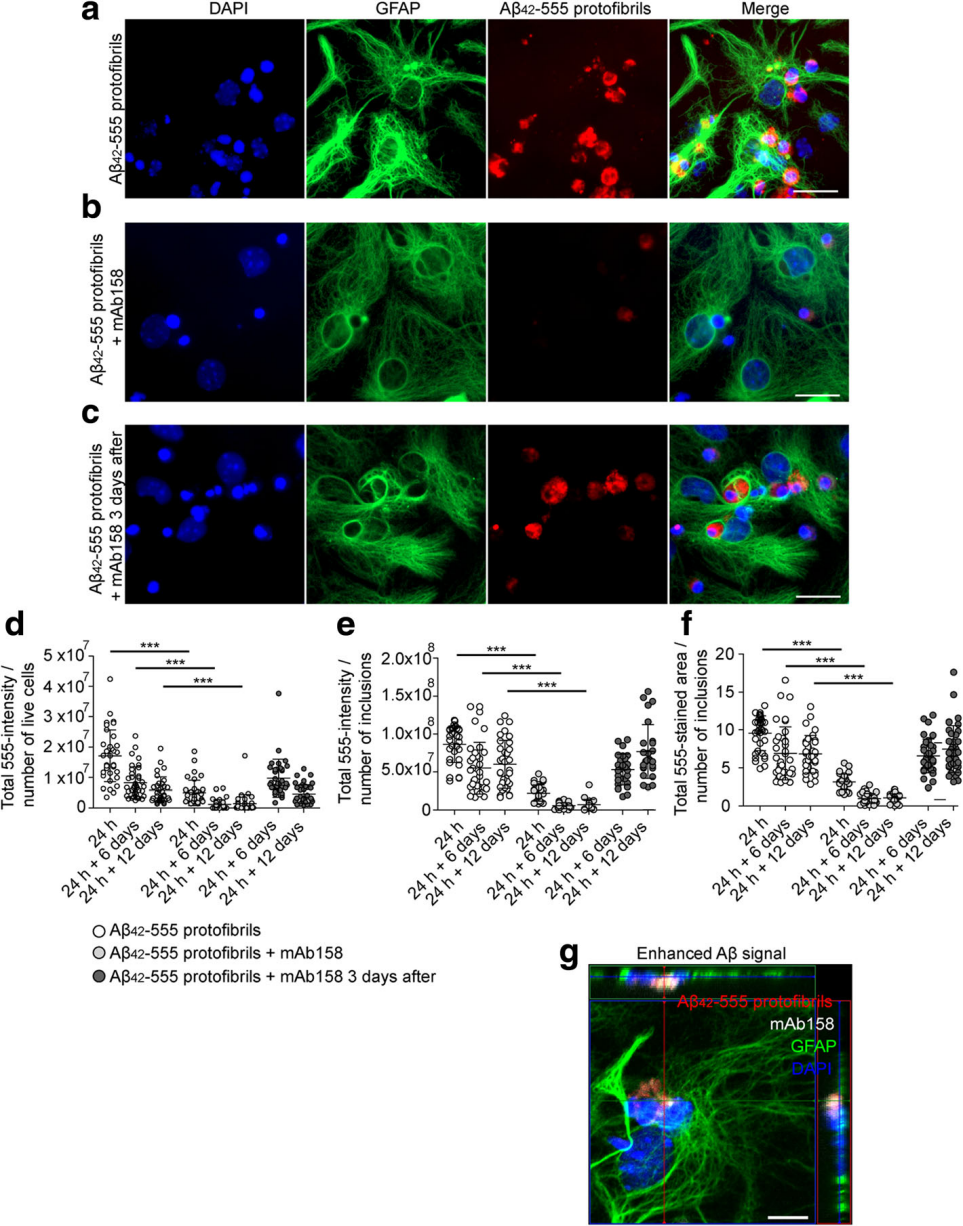
mAb158 reduces Aβ accumulation in astrocytes. Exposure of co-cultures to Aβ_42_-555 protofibrils for 24 h results in large Aβ inclusions in astrocytes (**a**). Concurrent addition of mAb158 clearly reduces the intracellular Aβ_42_-555 in astrocytes (**b**). However, mAb158 added 3 days after the Aβ_42_-555 protofibril exposure had no effect on Aβ_42_-555 accumulation (**c**). Using the Zen software, the 555-intensity and 555-stained area of the inclusions were measured. The 555-intensity/number of living cells (**d**), 555-intensity/number of Aβ inclusions (**e**), and total 555-stained area of Aβ-555 inclusions (**f**) decreased significantly in co-cultures treated with Aβ_42_-555 protofibrils + mAb158, compared to cultures exposed to Aβ_42_-555 protofibrils only or cultures treated with mAb158 3 days after Aβ_42_-555 protofibril exposure. Confocal imaging demonstrates intracellular co-localization of Aβ_42_-555 protofibrils and mAb158 in astrocyte (**g**). GFAP (green), DAPI (blue), mAb158 (white), and Aβ_42_-555 (red). The image is an orthogonal view of a confocal z-stack image. The XZ plane and YZ plane are shown at the top and to the right of the XY image, respectively. Scale bar 20 μm. The experiments were performed in triplicates with independent cell cultures, and 10 images/experiment were analyzed. Statistical analysis using Mann–Whitney *U* test (***P < 0.001)

To investigate if the Aβ protofibril selective antibody, mAb158, could increase Aβ clearance in astrocytes, primary cortical co-cultures containing neurons and glia were exposed to Aβ_42_-555 protofibrils or Aβ_42_-555 protofibrils + mAb158 for 24 h. The cell cultures were either fixed directly after exposure (24 h) or washed and cultured for an additional 6 (24 h + 6 days) or 12 (24 h + 12 days) days without treatment, prior to fixation. Immunocytochemistry against the astrocytic marker GFAP demonstrated that in co-cultures exposed to Aβ_42_-555 protofibrils, the astrocytes contained very large Aβ inclusions, as previously reported [31] (Fig. 1a). Preincubation of the Aβ_42_-555 protofibrils with mAb158 resulted in a dramatic reduction of the astrocytic Aβ inclusions, already at the first time point (Fig. 1b). To investigate if the Aβ protofibril selective antibody mAb158 could affect Aβ accumulation in astrocytes after the Aβ ingestion had taken place, cells were treated with mAb158 3 days after the 24 h Aβ_42_-555 protofibril exposure (mAb158 + 3 days). There was no evident effect on Aβ accumulation in mAb158 + 3-day-treated co-cultures compared to co-cultures exposed to Aβ_42_-555 protofibril exposure only (Fig. 1c). In addition, mAb158 added to co-cultures 1 h prior to the Aβ_42_ protofibril exposure had a reduced effect on astrocytic Aβ accumulation compared to mAb158 co-incubated with Aβ_42_ protofibrils (Additional file 2).

The total 555-intensity, normalized to the number of live cells (Fig. 1d), the number of inclusions (Fig. 1e), and the total area (Fig. 1f) of the intracellular Aβ-555 inclusions in the co-cultures were measured using the Zen 2012 software. The quantifications demonstrated that there was a significant reduction in 555-intensity and 555-stained area of the Aβ inclusions at 24 h + 6 days and 24 h + 12 days, when the antibody was present simultaneously with the Aβ_42_-555 protofibrils (*P* < 0.001 for 555-intensity/number of living cells, 555-intensity/number of Aβ inclusions and area of Aβ inclusions). When the mAb158 antibody was added 3 days after the Aβ_42_-555 protofibril exposure there was no difference in 555-intensity/number of living cells, 555-intensity/number of Aβ inclusions and area of Aβ inclusions as compared to co-cultures exposed to Aβ_42_-555 protofibrils only (Fig. 1d–f). Similar to mAb158, the presence of mAb1C3 (binding pan-Aβ) [42, 43] lowered the 555-intensity and 555-stained area of Aβ inclusions in the astrocytes, compared to Aβ_42_ protofibril-exposed astrocytes (Additional file 3). This result was expected since mAb1C3 binds to all forms of Aβ, including Aβ_42_ protofibrils. To investigate if the effect was specific for Aβ antibodies, co-cultures were exposed with Aβ_42_ protofibrils together with the irrelevant antibody Ly-128 (IgG_1_) or Aβ_42_ protofibrils together with the irrelevant antibody MOPC-173 (IgG_2a_). Ly-128 did not reduce the intracellular Aβ accumulation (Additional file 2) and MOPC-173 had a significantly lower effect on the Aβ accumulation, as compared to mAb158 (Additional file 4). In co-cultures treated with the irrelevant antibody MOPC-173, there was a threefold higher 555-intensity/live cells, a fivefold higher 555-intensity/number of inclusions, and a threefold larger area/number of 555-inclusions, compared to co-cultures treated with mAb158 (Additional file 4 D-E). The reason why there was some effect also with the MOPC-173 antibody is probably that Aβ is a very sticky protein and that the Aβ_42_ protofibrils to some degree bind unspecifically to the MOPC-173 antibody.

### mAb158 and Aβ_42_ protofibrils co-localize in astrocytes

To be able to follow uptake and accumulation over time, we performed time-lapse experiments of co-cultures exposed to Aβ_42_-555 protofibrils and mAb158 labeled with DyLight™ 488. The recordings of the co-cultures, 30 min to 24 h after addition demonstrated that the Aβ_42_-555 protofibrils and mAb158 were taken up and co-localized inside the astrocytes (identified by their phenotype of an egg “sunny side up”, large nuclei, and multi-vesicular cytoplasm). It should be noted that although some Aβ_42_-555 protofibril uptake was observed, the intracellular accumulation of Aβ was minute compared to controls that received Aβ_42_-555 protofibrils only (Additional file 5). Confocal microscopy of astrocytes exposed to Aβ_42_-555 protofibrils preincubated with mAb158 confirmed that low levels of intracellular Aβ_42_-555 protofibrils co-localized with mAb158 (Fig. 1g).

### mAb158 rescues neurons from Aβ-induced toxicity

**Fig. 2:**
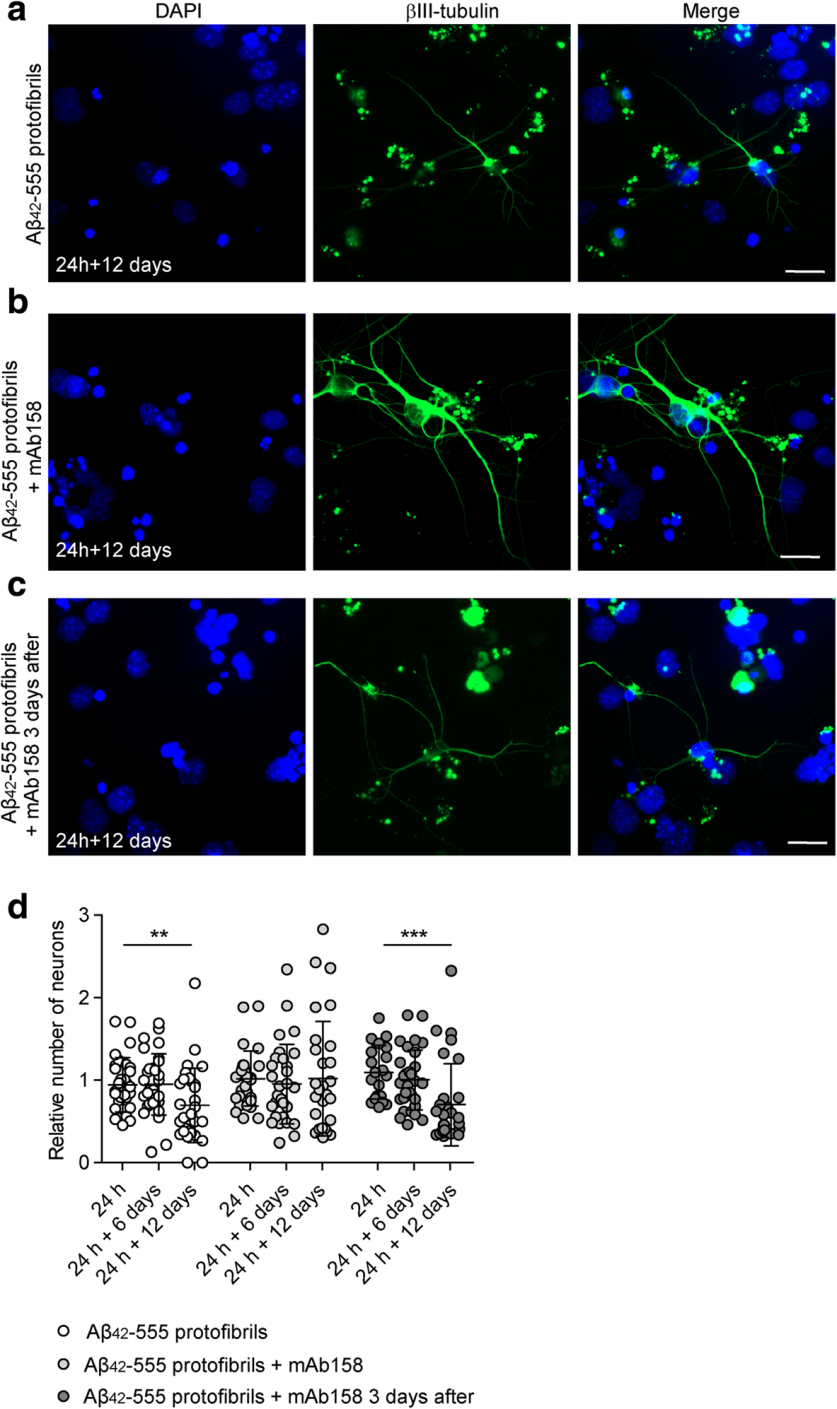
mAb158 rescues neurons from Aβ-induced toxicity. Aβ_42_-555 protofibril exposure of co-cultures results in neuronal cell death 12 days following the removal of the Aβ_42_-555 protofibrils (**a**). Simultaneous addition of mAb158, significantly rescued the neurons (**b**), but this effect was not seen when the mAb158 was added 3 days after the Aβ_42_-555 protofibrils (**c**). The relative number of living neurons was significantly decreased 12 days following Aβ_42_-555 protofibril exposure (24 h + 12 days, **P < 0.01), but no neurotoxic effect was observed at 24 h + 12 days in cultures exposed to Aβ_42_-555 protofibrils together with mAb158. When mAb158 were added 3 days after the Aβ_42_-555 protofibril removal, no protective effect of mAb158 was found (***P < 0.001) (**d**). βIII-tubulin (green) and DAPI (blue). Scale bar 20 μm. The experiments were performed in triplicates with independent cell cultures, and 10 images/experiment were analyzed using Mann–Whitney *U* test

We have previously shown that astrocytes exposed to Aβ_42_ protofibrils induce neuronal toxicity by secreting Aβ-containing microvesicles [31]. Hence, we sought to investigate whether mAb158 had any protective effect on Aβ_42_ protofibril-induced neuronal toxicity in the co-culture system. Aβ_42_-555 protofibrils, preincubated with mAb158, were added to the co-cultures for 24 h, 24 h + 6, or 24 h + 12 days. The co-cultures were stained for the neuronal marker βIII-tubulin and for the nuclear dye DAPI, and the number of living neurons was manually counted. As previously shown, Aβ_42_-555 protofibrils significantly decreased the neuronal viability 12 days after Aβ_42_-555 protofibril removal [31]. Interestingly, the presence of mAb158 rescued neurons from Aβ_42_-555 protofibril-induced toxicity at 24 h + 12 days. However, mAb158 addition 3 days after Aβ_42_-555 protofibril removal had no effect on the viability, and the number of neurons decreased significantly from 24 h to 24 h + 12 days (*P* < 0.0001) (Fig. 2).

### N297D, a mutated version of RmAb158 with reduced effector function, also lowers Aβ_42_ protofibril, but less effectively

**Fig. 3:**
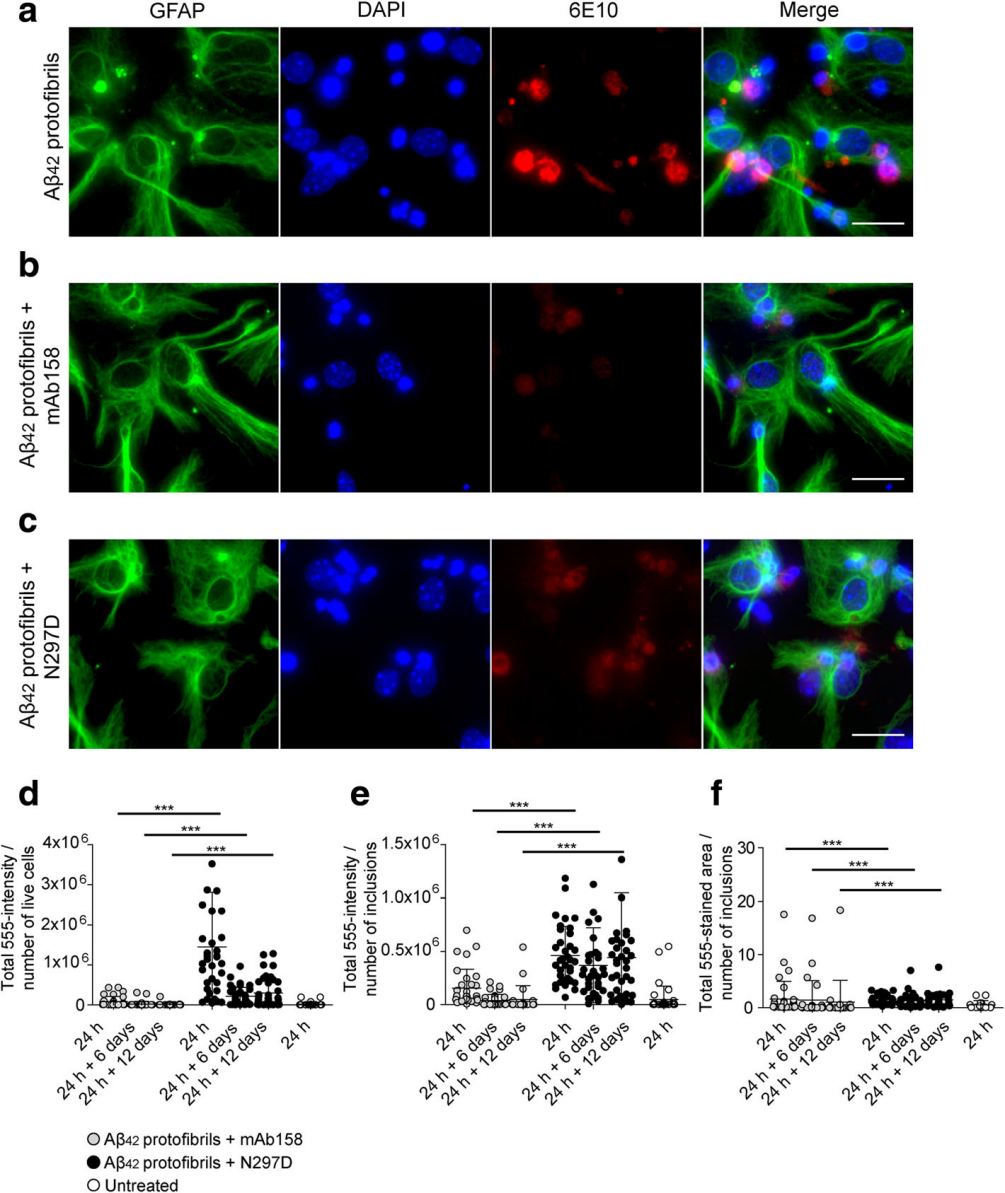
mAb158 reduces intracellular Aβ load, at least partly through an Fcγ receptor-independent pathway. Aβ_42_ protofibril accumulation in astrocytes (**a**) is reduced by preincubating Aβ_42_ protofibrils with mAb158 (**b**) or with N297D (**c**). Although, there was a clear reduction in Aβ accumulation with the presence of N297D as compared to Aβ_42_ protofibril exposed co-cultures, the cultures treated with mAb158 had significantly lower 555-intensity/number of living cells (**d**), 555-intensity/number of Aβ inclusions (**e**), and total 555-stained area of Aβ inclusions (**f**), compared to cultures treated with N297D. GFAP (green), DAPI (blue), and Aβ (red). Scale bar 20 μm. The experiments were performed in triplicates with independent cell cultures, and 10 images/experiment were analyzed. Statistical analysis using Mann–Whitney *U* test (***P < 0.001)

**Fig. 4:**
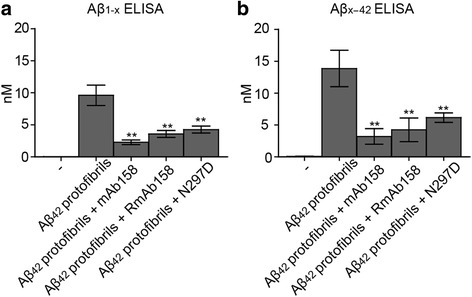
Anti-Aβ protofibril selective antibodies reduce the levels of intracellular Aβ. The Aβ levels in total cell lysates were measured using Aβ_1-x_ and Aβ_x-42_ ELISA. Both Aβ_1-x_ (**a**) and Aβ_x-42_ (**b**) levels were reduced in cultures treated with Aβ_42_ protofibrils + mAb158, Aβ_42_ protofibrils + RmAb158, or Aβ_42_ protofibrils + N297D, compared to cultures exposed to Aβ_42_ protofibrils only. All concentrations are expressed in nanomolar (nM) units. Mean values are from duplicates of three independent experiments, from two repeated analyses (**P < 0.01)

Next, we sought to investigate if the mAb158 effect on Aβ_42_ protofibril accumulation was Fcγ receptor dependent or independent. For this purpose, we used the N297D antibody, a recombinantly produced mAb158 antibody (RmAb158) with a mutation in the glycosylation site, leading to loss of Fcγ receptor binding and reduced C1q binding. Both N297D and RmAb158 have the same antigen recognition site as the hybridoma-produced mAb158. Immunocytochemistry of fixed co-cultures exposed to Aβ_42_ protofibrils and N297D showed a similar reduction of Aβ accumulation in astrocytes as mAb158 after 24 h (Fig. 3a–c). The 555-intensity and 555-stained area of the intracellular Aβ were measured by the Zen 2012 software. N297D treatment decreased the 555-staining intensity significantly already at 24 h. Although a treatment effect was obtained with N297D, mAb158-treated cultures had significantly lower total 555-intensity per number of living cells and number of the inclusions, and the 555-stained area of the inclusions was significantly smaller (Fig. 3d–f). As mAb158, RmAb158, a suitable control to N297D, lowered the intracellular Aβ inclusions in astrocytes (Additional file 6). To further study the effect on intracellular Aβ levels in co-cultures exposed to the different treatments, Aβ_1-x_ and Aβ_x-42_ levels were measured by ELISA in cell lysates after 24 h Aβ_42_ protofibril or Aβ_42_ protofibril + mAb158, Aβ_42_ protofibril + RmAb158 or Aβ_42_ protofibril + N297D treatment. The levels of Aβ_1-x_ (9614 ± 3878 pM) and Aβ_x-42_ (13,845 ± 2861 pM) were markedly reduced upon preincubation of Aβ_42_ protofibrils with mAb158 (Aβ_1-x_ 2282 ± 871 pM, Aβ_x-42_ 3845 ± 1234 pM) and RmAb158 (Aβ_1-x_ 3577 ± 1338 pM, Aβ_x-42_ 4238 ± 1851 pM). A reduction of Aβ_1-x_ and Aβ_x-42_ levels was also observed for N297D (Aβ_1-x_ 4251 ± 1329 pM, Aβ_x-42_ 6153 ± 739 pM) (Fig. 4a, b).

**Fig. 5:**
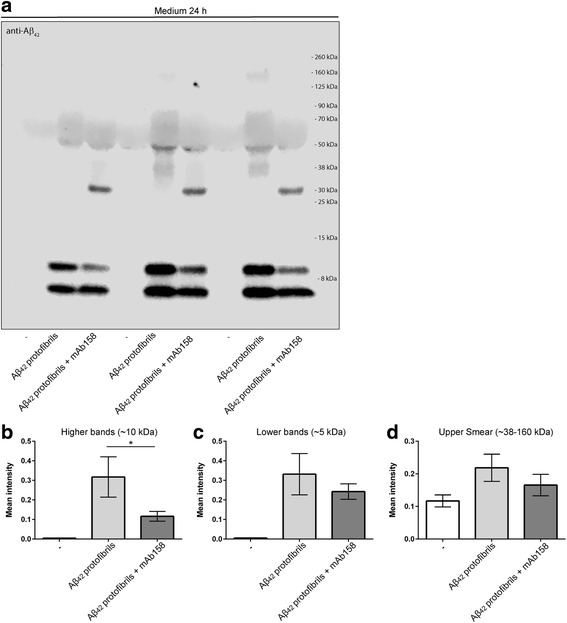
In the presence of mAb158, Aβ is cleared from the cell culture media. Western blot analysis showed a reduction of Aβ in the cell culture media of cultures exposed to Aβ_42_ protofibrils + mAb158, compared to cultures exposed to Aβ_42_ protofibril only (**a**). The reduction was seen both in low-molecular Aβ at ~ 10 kDa (**b**) and ~ 5 kDa (**c**) and in the smear of Aβ detected at ~ 38–160 kDa (**d**). Intensity measurements were performed from three independent cell culture experiments, and statistical analysis was performed using one-way ANOVA followed by Tukey’s multiple comparison test (*P < 0.05)

To elucidate the extracellular levels of Aβ_42_, we performed Western blot analysis on the cell culture media from non-exposed and Aβ_42_ protofibril-exposed co-cultures with or without co-incubation of mAb158 for 24 h. A reduction of Aβ_42_ was observed in the presence of mAb158, compared to the Aβ_42_ protofibril-exposed co-cultures (Fig. 5a). Detection of low-molecular Aβ_42_ was found at both ~ 10 kDa (Fig. 5b) and ~ 5 kDa (Fig. 5c) and a smear of Aβ_42_ was detected from ~ 38–160 kDa (Fig. 5d). In all three Aβ fractions, the Aβ_42_ levels were decreased in mAb158-treated co-cultures. This effect was significant for the ~ 10 kDa dimer band, which is most likely a result of incomplete SDS-denaturing of the larger Aβ_42_ aggregates that the mAb158 selectively binds to. These results indicate that there is an increased clearance of Aβ_42_ protofibrils from the cell culture media in the presence of mAb158. The unknown band appearing at ~ 30 kDa in the cell culture media of mAb158-treated co-cultures could be due to unspecific binding of the primary antibody or cross-reactivity of the mAb158 light chain and the secondary antibody. Blotting the filter with a secondary mouse IgG antibody showed that both the heavy and light chain of the mAb158 antibody can be detected in the media of antibody-treated cultures (Additional file 7). Most likely the mAb158 antibody is bound to Aβ that remains in the media at this time point and has not yet been cleared by the cells.

### mAb158 F(ab’)_2_ reduces Aβ accumulation in astrocytes

**Fig. 6:**
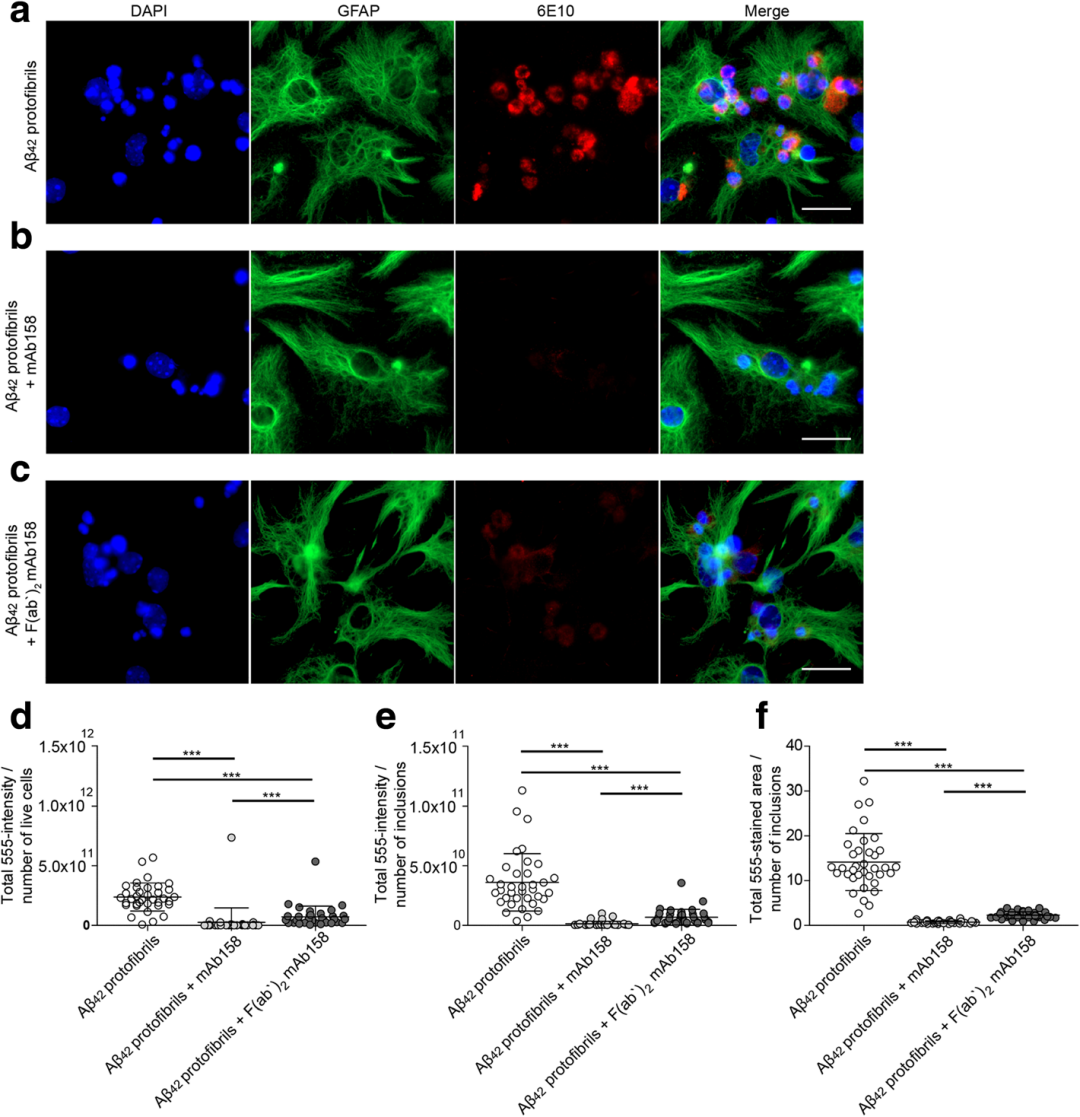
mAb158 F(ab’)_2_ fragments reduce Aβ accumulation in astrocytes. To confirm that the effect of mAb158 on Aβ_42_ protofibril degradation was a Fcγ receptor-independent mechanism, cell cultures were exposed to the F(ab’)_2_ fragments of mAb158 together with the Aβ_42_ protofibrils. Astrocytes in co-cultures exposed to Aβ_42_ protofibrils accumulated Aβ (**a**) but not when co-treated with mAb158 (**b**). In cultures treated with F(ab’)_2_ mAb158 + Aβ_42_ protofibrils, the accumulation of Aβ was reduced, almost to the same level as for mAb158 (**c**). The 555-intensity/number of living cells (**d**), 555-intensity/number of Aβ inclusions (**e**), and total 555-stained area of Aβ inclusions (**f**) were all significantly reduced in co-cultures treated with Aβ_42_ protofibril + mAb158 and Aβ_42_ protofibril + mAb158 F(ab’)_2_ fragments, as compared to Aβ_42_ protofibril exposed co-cultures. GFAP (green), DAPI (blue), and Aβ (red). Scale bar 20 μm. The experiments were performed in triplicates with independent cell cultures, and 10 images/experiment were analyzed using Mann–Whitney *U* test (***P < 0.001)

To confirm that the effect of mAb158 on Aβ_42_ protofibril degradation was an Fcγ receptor-independent mechanism, the F(ab’)_2_ fragments of mAb158 were co-incubated with Aβ_42_ protofibrils. The F(ab’)_2_ fragment lacks its Fc domain and will therefore not bind to the Fcγ receptor on the cells. Similar to mAb158, co-cultures exposed to F(ab’)_2_ mAb158 + Aβ_42_ protofibrils for 24 h reduced the astrocytic accumulation of Aβ (Fig. 6). Taken together, our data indicate that the effect of mAb158 on Aβ_42_ protofibril degradation occurs mainly through Fcγ receptor-independent mechanisms.

### Proteosomal or endosomal-lysosomal inhibitors do not alter mAb158-mediated effects

**Fig. 7:**
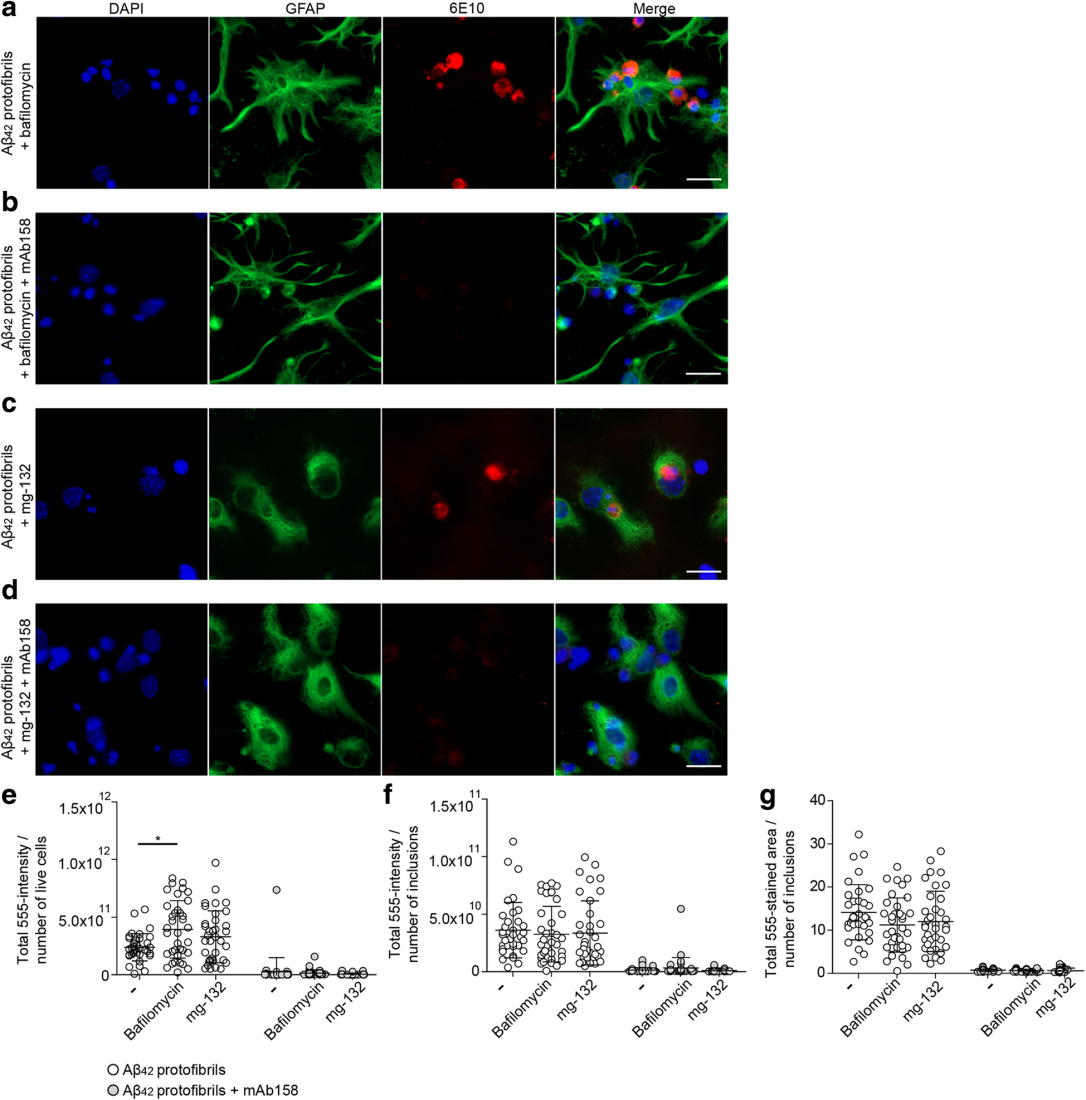
Proteosomal or endosomal-lysosomal inhibitors do not alter the mAb158-mediated effects on Aβ accumulation in astrocytes. Co-cultures were incubated with the proteosomal inhibitor mg-132 or the lysosomal inhibitor bafilomycin for 30 min prior to the Aβ_42_ protofibril exposure and mAb158 treatment. The inhibitors were also present during the 24-h exposure. Neither of the inhibitors affected the mAb158-mediated reduction of Aβ deposits (**a**–**d**). Measurement of 555-intensity/number of living cells (**e**), 555-intensity/number of Aβ inclusions (**f**), and total 555-stained area of Aβ inclusions (**g**) demonstrated that there was a significant reduction in Aβ inclusions in Aβ_42_ protofibril-exposed cultures when co-treated with mAb158 + bafilomycin or mAb158 + mg-132. This confirms that the inhibitors did not interfere with the action of mAb158. GFAP (green), DAPI (blue), and Aβ (red). Scale bar 20 μm. The experiments were performed in triplicates with independent cell cultures, and 10 images/experiment were analyzed using Mann–Whitney *U* test (**P < 0.01)

In order to investigate the influence of the proteosomal and endosomal-lysosomal pathway on mAb158-mediated Aβ reduction in astrocytes, we preincubated co-cultures with the proteosomal inhibitor mg-132 or the lysosomal inhibitor bafilomycin for 30 min prior to Aβ_42_ protofibril exposure ± mAb158 treatment. The inhibitors were also present during the 24-h exposure. Neither mg-132 nor Bafilomycin had any clear effect on the mAb158-mediated reduction of Aβ deposits in astrocytes (Fig. 7a–d). Measurement of the 555-intensity/number of living cells (Fig. 7e), 555-intensity/number of Aβ inclusions (Fig. 7f), and 555-stained area of Aβ inclusions (Fig. 7g) demonstrated that there was a significant reduction in Aβ at all the time points, confirming that the inhibitors did not interfere with the action of mAb158.

## Discussion

Accumulation of Aβ is a main event in AD pathology. Since the majority of the patients with sporadic, late-onset, AD do not have an increased Aβ production, insufficient lysosomal degradation has been suggested to be the most common cause of the disease for these patients [44–46]. Immunotherapy has emerged as a promising method to target Aβ pathology, and numerous antibodies directed towards different Aβ species are presently evaluated in clinical trials as therapeutics for AD. These antibodies may enhance clearance and/or prevent aggregation of Aβ, but the underlying mechanisms and the role of different cell types in Aβ-directed immunotherapy are still poorly understood. Here, we demonstrate for the first time that the Aβ protofibril selective monoclonal antibody mAb158 [47] increases clearance of pathological Aβ by astrocytes and thereby rescues neurons from secondary cell death.

Recently, BAN2401 [48], the humanized version of mAb158, was developed in a collaboration between BioArctic AB and Eisai and is currently evaluated in a phase 2b clinical trial [49]. Although mAb158 has been shown to reduce brain levels of Aβ protofibrils and prevent Aβ deposition in mouse models of AD [16, 48], the cellular mechanism of its action has not been elucidated.

We have previously demonstrated that astrocytes efficiently engulf Aβ_42_ protofibrils, but then store rather than degrade the ingested aggregates [31]. The aim of the present study was to investigate if the astrocytes’ potency to clear Aβ_42_ protofibrils could be modulated by the presence of mAb158. Our results demonstrate that mAb158 indeed affects the accumulation of Aβ in astrocytes, but that Aβ_42_ protofibrils and mAb158 need to be administered at the same time to enhance Aβ_42_ protofibril clearance, suggesting that the antibody must form a complex with Aβ to have an effect. Antibodies added to the cultures 3 days after the Aβ_42_ protofibril exposure had no effect on Aβ clearance.

In the presence of Aβ antibodies, phagocytic cells may clear Aβ through Fcγ receptor-mediated phagocytosis [50, 51]. The Fc region is highly sensitive to the presence of glycosylation at a single *N*-linked glycosylation site at asparagine 297 (N297), with a loss of binding to the low-affinity Fcγ receptors and diminished activation of the complement pathway observed in N297 point mutants [52–54]. Interestingly, we found that mAb158 containing a N297D mutation (Asparagine replaced by Aspartic acid) showed almost as good Aβ clearing effect in the astrocytes, as the mAb158 without mutation. To fully elucidate whether the effect of mAb158 on Aβ_42_ protofibril degradation was a Fcγ receptor-dependent or Fcγ receptor-independent mechanism, we studied the effect of a F(ab’)_2_ fragment of mAb158. The F(ab’)_2_ fragment lacks its Fc region and can therefore not bind to any of the Fcγ receptors. Our data showed F(ab’)_2_ mAb158 also significantly reduced the accumulation of Aβ in astrocytes, indicating that the effect of mAb158 on Aβ_42_ protofibril degradation is mainly a Fcγ receptor-independent mechanism. Our results go well in line with studies performed by Leabman et al. [55], where deglycosylation of antibodies reduced Fcγ receptor binding, but did not affect pharmacokinetics of the antibody compared to the wildtype antibody [56]. Moreover, observations in transgenic AD mice show that Aβ can be cleared through Fcγ receptor-independent mechanisms after treatment with F(ab’)_2_ fragments lacking the Fc region and after anti-Aβ immunization in Fcγ receptor ^−/−^ knockout mice [57, 58].

Our results show that inhibition of the lysosomal or proteosomal pathway did not have an impact on the reduced accumulation, possibly indicating that the majority of the Aβ antibody-Aβ_42_ protofibril complexes may be degraded by another, not yet described mechanism. An explanation for the reduced intracellular deposits of Aβ could be a lower uptake or an increased recycling and secretion of the engulfed Aβ antibody-Aβ_42_ protofibril complexes. However, since astrocytes engulf larger particles by macropinocytosis [59], a non-selective engulfment in which the cells take up large gulps of the surrounding liquid (and free-floating particles), the uptake cannot be blocked by the presence of an antibody. Moreover, our ELISA and Western blot analyses of lysates and conditioned media showed that both the intra- and extracellular levels of Aβ were lowered in the antibody-treated cultures. In accordance, the time-lapse recordings indicated a treatment-related decrease in the astrocytic accumulation and absence of free-floating Aβ antibody-Aβ_42_ protofibril complexes. The reason why the addition of mAb158 after Aβ_42_ protofibril exposure (and washes) had no effect, is probably due to the fact that no Aβ remains in the medium, but is situated intracellularly, in endosomal compartments [31] and might not be easily accessible by the antibodies.

## Conclusion

In conclusion, our data demonstrate that the Aβ antibody-Aβ_42_ protofibril complexes do not accumulate in the astrocytes to the same extent as Aβ_42_ protofibrils alone, but are rather cleared from the co-culture. This is in line with our previous data, demonstrating that alpha-synuclein selective antibodies promote clearance of both intracellular and extracellular alpha-synuclein aggregates [59, 60].We have previously reported that accumulation of Aβ_42_ protofibrils by astrocytes result in increased neurotoxicity, due to secretion of microvesicles containing truncated Aβ_42_ [31]. Interestingly, enhanced Aβ degradation in astrocytes, treated with mAb158, protected neurons from Aβ_42_ protofibril-induced toxicity.

To enable development of new therapeutic targets for AD, increased understanding of the pathways behind engulfment and intracellular degeneration of Aβ is necessary. In order to optimize the design of future Aβ antibody-based therapies, we believe that more studies are needed, focusing on various cell types, including astrocytes.

## Additional files

Additional file 1: Iba-1 immunostaining confirms the absence of microglia in the co-cultures. Immunocytochemistry, with a specific antibody to Iba-1, were performed to verify that no microglia were present in the co-cultures (A). A brain tissue section from a 16-month-old APP_ArcSwe_ mouse was included as a positive control (B). Microglia were not detected in the co-cultures, but were frequently found in the positive control. Scale bars: 20 μm. (TIFF 4932 kb)

Additional file 2: The irrelevant antibody Ly-128 does not reduce the intracellular Aβ accumulation. The Aβ aggregates that were formed in astrocytes in Aβ_42_ protofibril exposed cultures (A) were clearly reduced in the presence of mAb158 (B). To ensure that this effect was specific for Aβ antibodies, mAb158 was exchanged to the irrelevant antibody Ly-128 (IgG_1_) (C). Ly-128 did not reduce the intracellular Aβ accumulation. In addition, mAb158 had only a minor effect on the Aβ accumulation if it was added to the co-cultures for 1 h prior to the Aβ_42_ protofibril exposure (D). mAb158 had a significantly higher effect on the Aβ accumulation, compared to mAb158 (1 h). The total 555-intensity was analyzed per number of live cells (E) and number of inclusions (F), and the total 555-stained area per number of inclusions (G) significantly decreased when treated with mAb158 compared to Aβ_42_ protofibril or Aβ_42_ protofibril + Ly-128 exposed co-cultures. GFAP (green), DAPI (blue), Aβ_42_ (red). Scale bar: 20 μm. The experiments were performed in triplicates with independent cell cultures and 10 images/experiment were analyzed using Mann-Whitney *U*-test (**P < 0.01 and ***P < 0.001). (TIFF 5989 kb)

Additional file 3: The mAb1C3 lowers Aβ inclusions in astrocytes. Aβ_42_ protofibrils were accumulated in astrocytes (A), but addition of the mAb1C3, binding pan-Aβ, to the co-cultures lowered the accumulation of Aβ_42_ protofibrils (B). The total 555-intensity was analyzed per number of live cells (C) and number of inclusions (D), and the total 555-stained area per number of inclusions (E). Taken together, the analyses confirmed that mAb1C3 lowers Aβ_42_ inclusions in astrocytes. Phalloidin (green), DAPI (blue), Aβ_42_ (red). Scale bar: 20 μm. The experiments were performed in triplicates with independent cell cultures and 10 images/experiment were analyzed using Mann-Whitney *U*-test (***P < 0.001). (TIFF 6026 kb)

Additional file 4: The irrelevant antibody MOPC-173 has a significantly lower effect on Aβ accumulation in astrocytes than mAb158. Aβ_42_ protofibrils were accumulated in astrocytes (A), and addition of mAb158 to the co-cultures lowered the accumulation of Aβ_42_ protofibrils (B). Addition of the irrelevant antibody MOPC-173 partly lowered the Aβ_42_ accumulation in astrocytes (C). The total 555-intensity was analyzed per number of live cells (D) and number of inclusions (E), and the total 555-area per number of inclusions (F). Taken together, the analysis shows that mAb158 had a significantly higher effect on the Aβ accumulation, compared to MOPC-173. Phalloidin (green), DAPI (blue), Aβ_42_ (red). Scale bar: 20 μm. The experiments were performed in triplicates with independent cell cultures and 10 images/experiment were analyzed using Mann-Whitney *U*-test (**P < 0.01 and ***P < 0.001). (TIFF 5931 kb)

Additional file 5: Aβ_42_-555 protofibrils and mAb158 are engulfed and co-localize inside astrocytes. Time-lapse recording demonstrating co-localization between Aβ_42_-555 protofibrils and DyLight™ 488 labeled mAb158 antibody in astrocytes (A). However, in the presence of antibodies, Aβ_42_-555 protofibrils reached much weaker signals, compared to cultures exposed to Aβ_42_-555 protofibrils only (B). Scale bars: A and B = 10 μm. (TIFF 6980 kb)

Additional file 6: RmAb158 reduces Aβ inclusions in astrocytes. Co-cultures were exposed to Aβ_42_ protofibrils (A) or Aβ_42_ protofibrils together with RmAb158 (IgG_2c_) (B). Measurements of the total 555-intensity per number of live cells (C) and number of inclusions (D), and the total 555-area per number of inclusions (E) confirmed that RmAb158 reduces Aβ inclusions in astrocytes. GFAP (green), DAPI (blue), Aβ (red). Scale bar: 20 μm. The experiments were performed in triplicates with independent cell cultures and 10 images/experiment were analyzed using Mann-Whitney *U*-test (***P < 0.001). (TIFF 4151 kb)

Additional file 7: The heavy and light chain of the mAb158 antibody can be detected in the media. Reprobing the filter in Fig. 5 with a secondary anti-mouse IgG antibody, showed that both the heavy and light chain of the mAb158 antibody can be detected in the media of antibody-treated cultures. (TIFF 2941 kb)

## Abbreviations

AD: Alzheimer’s disease
Aβ: Amyloid beta
Aβ_42_-555: Fluorescent HiLyte™ Fluor 555-labeled Aβ_42_
BSA: Bovine serum albumin
DAPI: 4′,6-Diamidino-2-phenylindole
E14: Embryonal day 14
ECL: Enhanced chemiluminescence
GFAP: Glial fibrillary acidic protein
N297: *N*-linked glycosylation site at asparagine 297
NGS: Normal goat serum
PBS: Phosphate-buffered saline
RmAb158: Recombinant mAb158
SDS: Sodium dodecyl sulfate
