# Supplementary figures and images for: The Aβ protofibril selective antibody mAb158 prevents accumulation of Aβ in astrocytes and rescues neurons from Aβ-induced cell death
Source: J Neuroinflammation. 2018 Mar 28;15:98. doi: 10.1186/s12974-018-1134-4 (PMC5875007; doi:10.1186/s12974-018-1134-4)

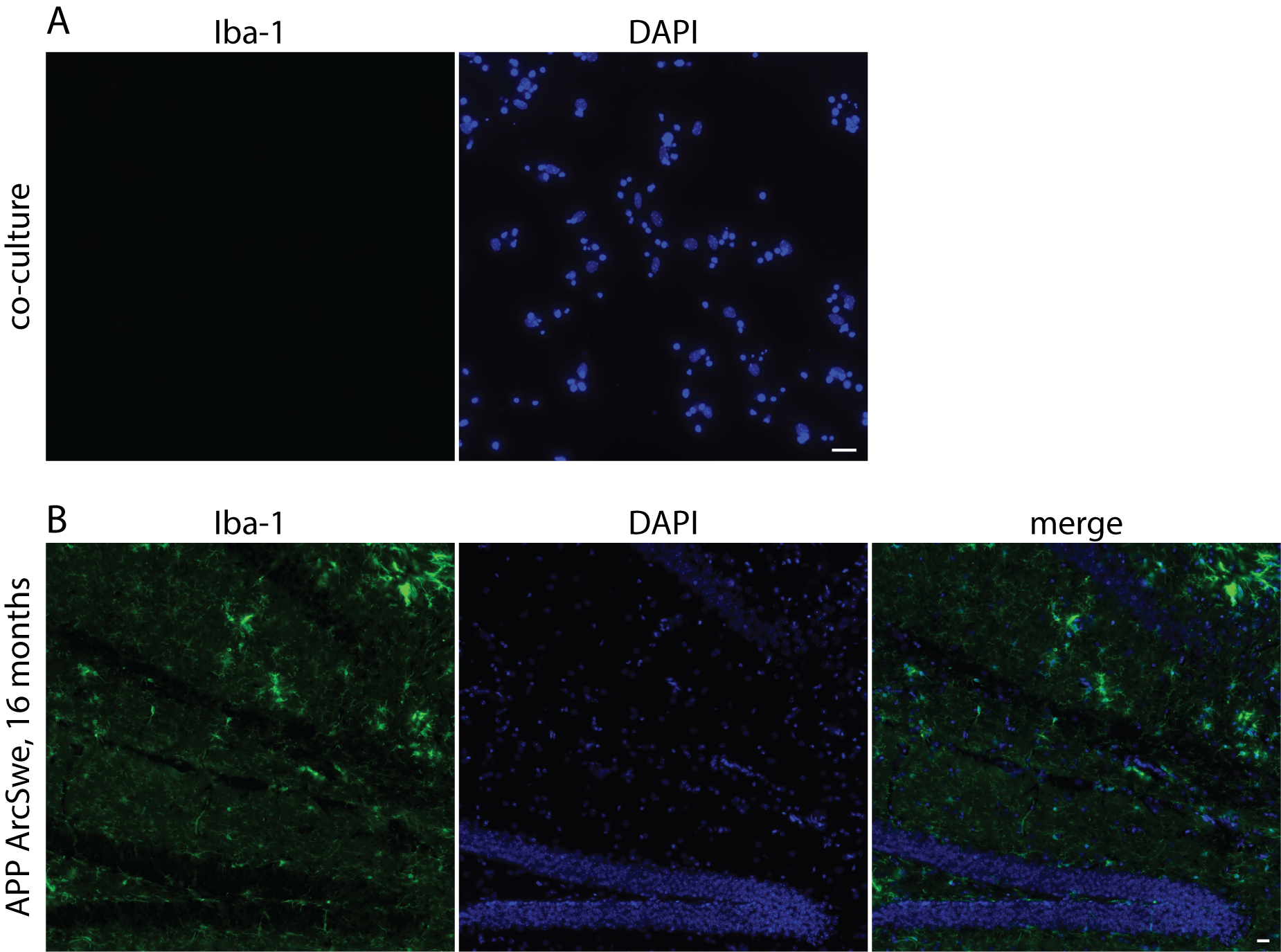

Supplement: Supplementary file 1 — Iba-1 immunostaining confirms the absence of microglia in the co-cultures. Immunocytochemistry, with a specific antibody to Iba-1, were performed to verify that no microglia were present in the co-cultures (A). A brain tissue section from a 16-month-old APPArcSwe mouse was included as a positive control (B). Microglia were not detected in the co-cultures, but were frequently found in the positive control. Scale bars: 20 μm. (TIFF 4932 kb) [file 12974_2018_1134_MOESM1_ESM.tif]

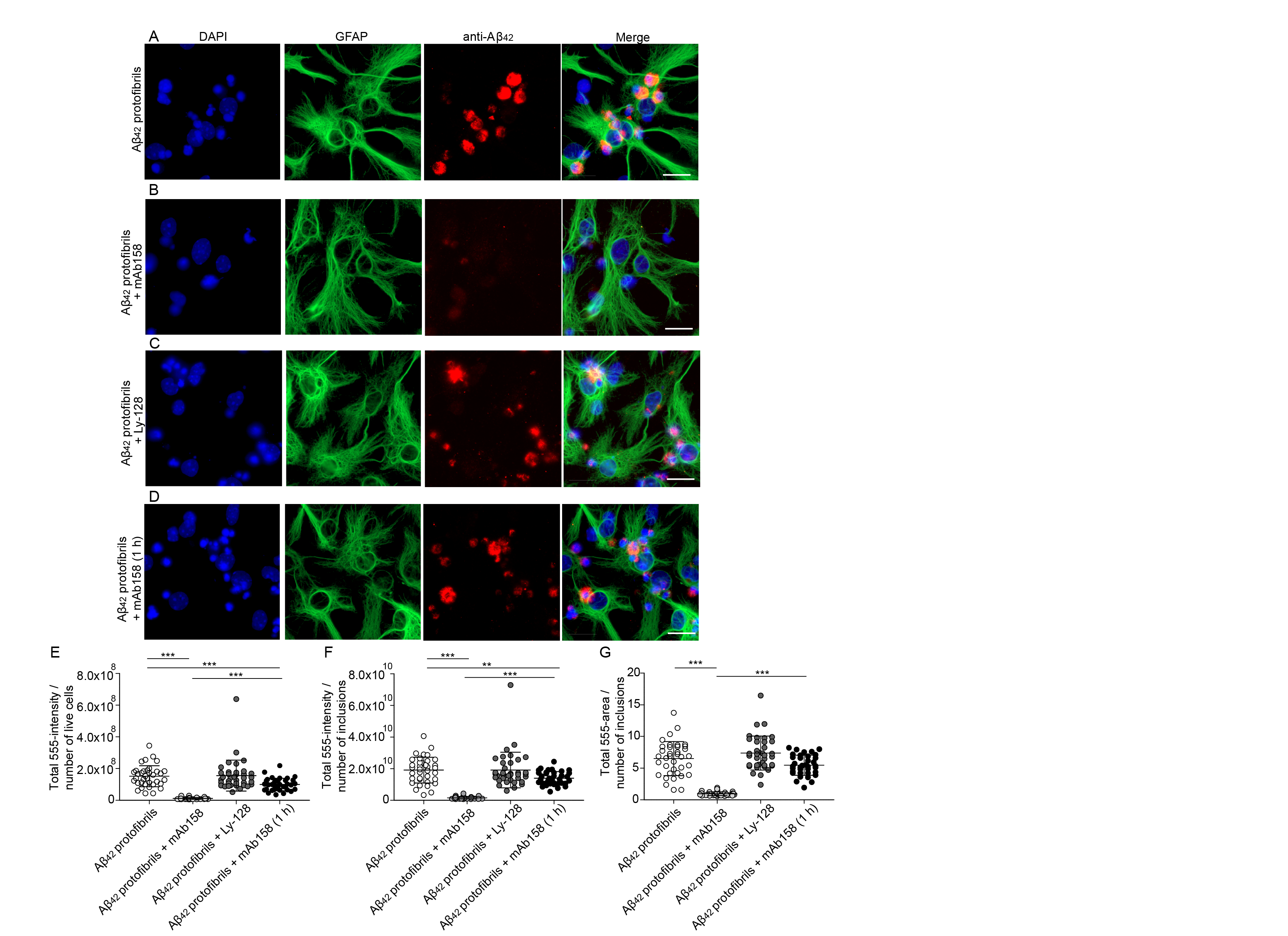

Supplement: Supplementary file 2 — The irrelevant antibody Ly-128 does not reduce the intracellular Aβ accumulation. The Aβ aggregates that were formed in astrocytes in Aβ42 protofibril exposed cultures (A) were clearly reduced in the presence of mAb158 (B). To ensure that this effect was specific for Aβ antibodies, mAb158 was exchanged to the irrelevant antibody Ly-128 (IgG1) (C). Ly-128 did not reduce the intracellular Aβ accumulation. In addition, mAb158 had only a minor effect on the Aβ accumulation if it was added to the co-cultures for 1 h prior to the Aβ42 protofibril exposure (D). mAb158 had a significantly higher effect on the Aβ accumulation, compared to mAb158 (1 h). The total 555-intensity was analyzed per number of live cells (E) and number of inclusions (F), and the total 555-stained area per number of inclusions (G) significantly decreased when treated with mAb158 compared to Aβ42 protofibril or Aβ42 protofibril + Ly-128 exposed co-cultures. GFAP (green), DAPI (blue), Aβ42 (red). Scale bar: 20 μm. The experiments were performed in triplicates with independent cell cultures and 10 images/experiment were analyzed using Mann-Whitney U-test (**P < 0.01 and ***P < 0.001). (TIFF 5989 kb) [file 12974_2018_1134_MOESM2_ESM.tif]

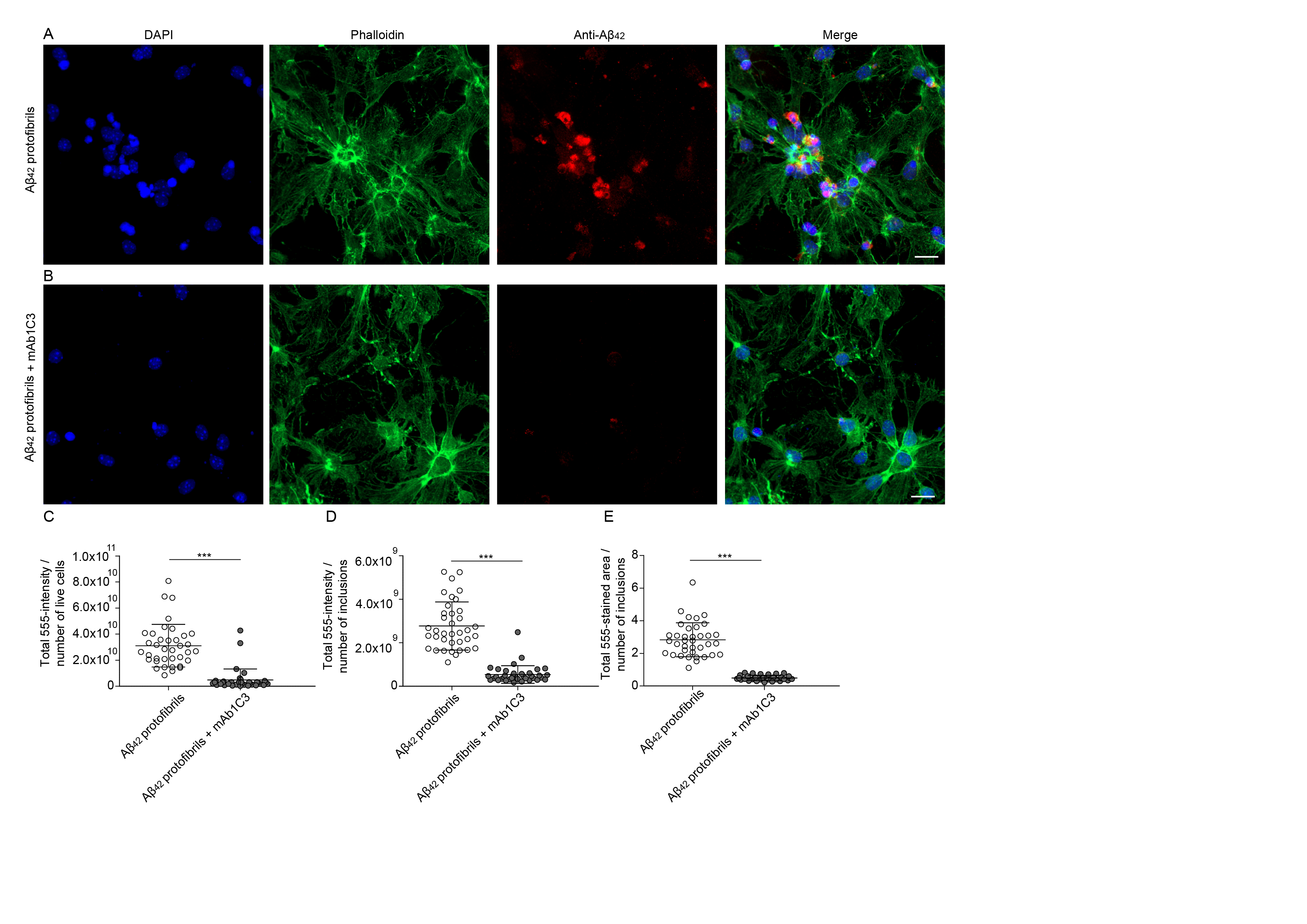

Supplement: Supplementary file 3 — The mAb1C3 lowers Aβ inclusions in astrocytes. Aβ42 protofibrils were accumulated in astrocytes (A), but addition of the mAb1C3, binding pan-Aβ, to the co-cultures lowered the accumulation of Aβ42 protofibrils (B). The total 555-intensity was analyzed per number of live cells (C) and number of inclusions (D), and the total 555-stained area per number of inclusions (E). Taken together, the analyses confirmed that mAb1C3 lowers Aβ42 inclusions in astrocytes. Phalloidin (green), DAPI (blue), Aβ42 (red). Scale bar: 20 μm. The experiments were performed in triplicates with independent cell cultures and 10 images/experiment were analyzed using Mann-Whitney U-test (***P < 0.001). (TIFF 6026 kb) [file 12974_2018_1134_MOESM3_ESM.tif]

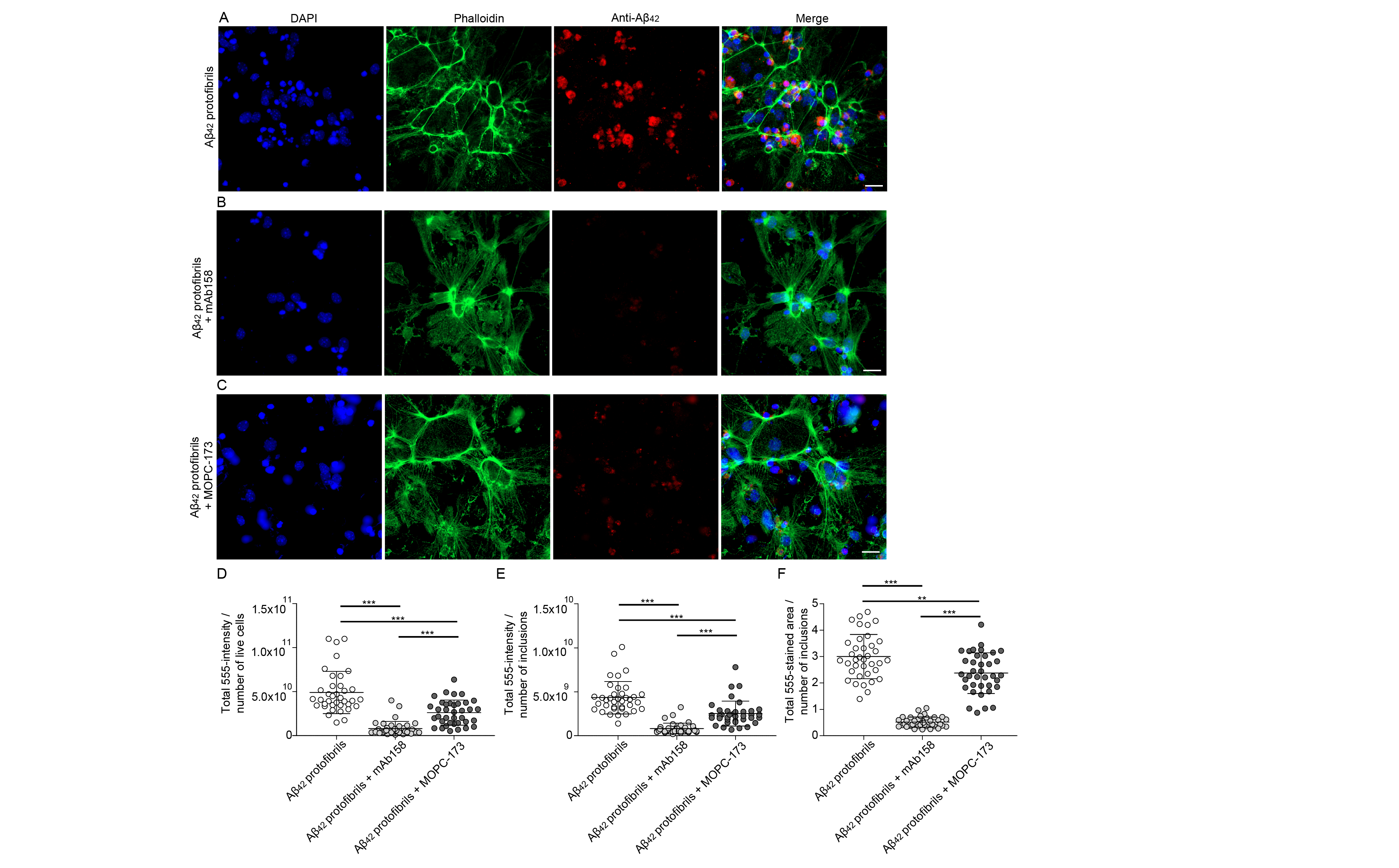

Supplement: Supplementary file 4 — The irrelevant antibody MOPC-173 has a significantly lower effect on Aβ accumulation in astrocytes than mAb158. Aβ42 protofibrils were accumulated in astrocytes (A), and addition of mAb158 to the co-cultures lowered the accumulation of Aβ42 protofibrils (B). Addition of the irrelevant antibody MOPC-173 partly lowered the Aβ42 accumulation in astrocytes (C). The total 555-intensity was analyzed per number of live cells (D) and number of inclusions (E), and the total 555-area per number of inclusions (F). Taken together, the analysis shows that mAb158 had a significantly higher effect on the Aβ accumulation, compared to MOPC-173. Phalloidin (green), DAPI (blue), Aβ42 (red). Scale bar: 20 μm. The experiments were performed in triplicates with independent cell cultures and 10 images/experiment were analyzed using Mann-Whitney U-test (**P < 0.01 and ***P < 0.001). (TIFF 5931 kb) [file 12974_2018_1134_MOESM4_ESM.tif]

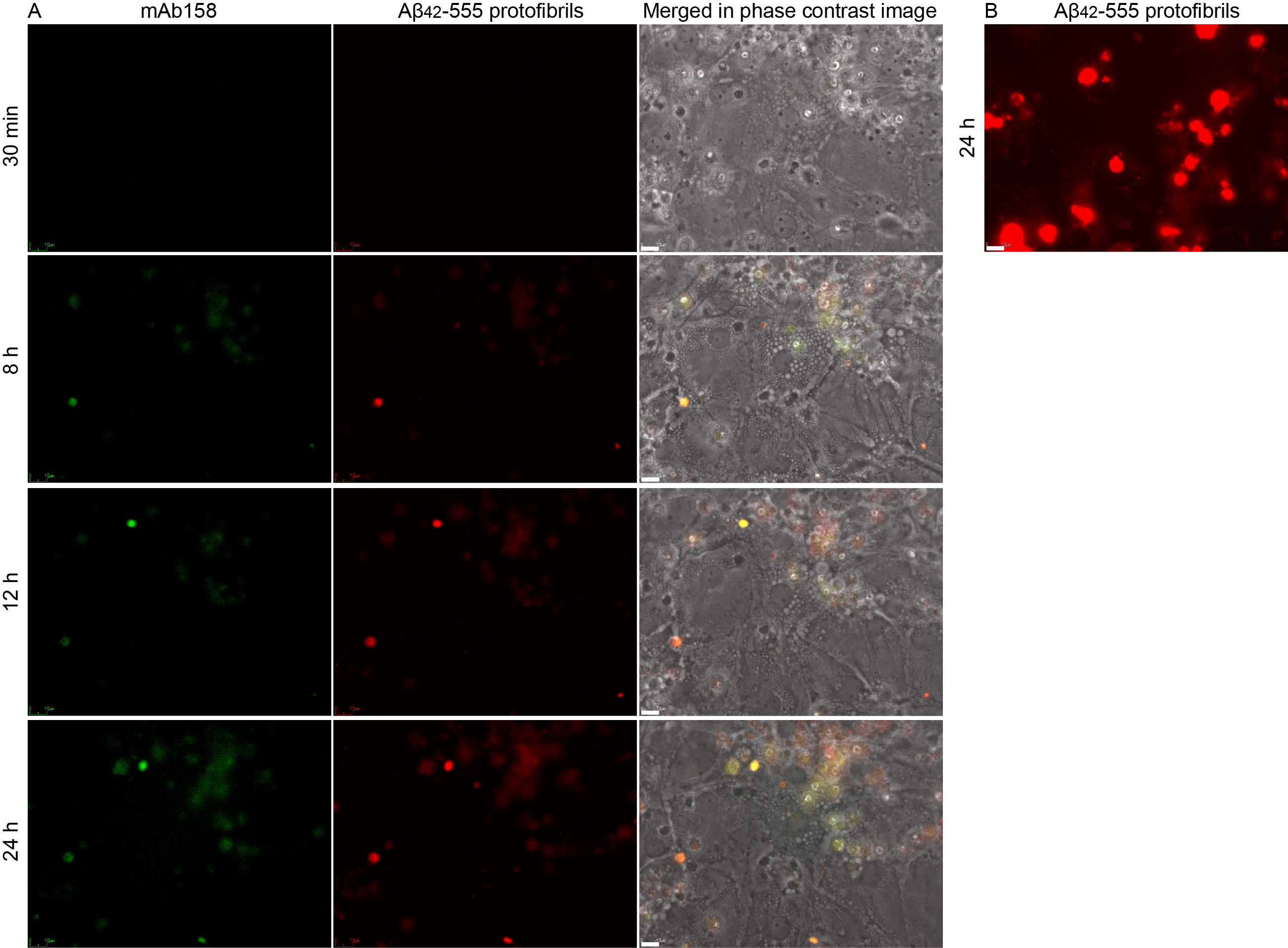

Supplement: Supplementary file 5 — Aβ42-555 protofibrils and mAb158 are engulfed and co-localize inside astrocytes. Time-lapse recording demonstrating co-localization between Aβ42-555 protofibrils and DyLight™ 488 labeled mAb158 antibody in astrocytes (A). However, in the presence of antibodies, Aβ42-555 protofibrils reached much weaker signals, compared to cultures exposed to Aβ42-555 protofibrils only (B). Scale bars: A and B = 10 μm. (TIFF 6980 kb) [file 12974_2018_1134_MOESM5_ESM.tif]

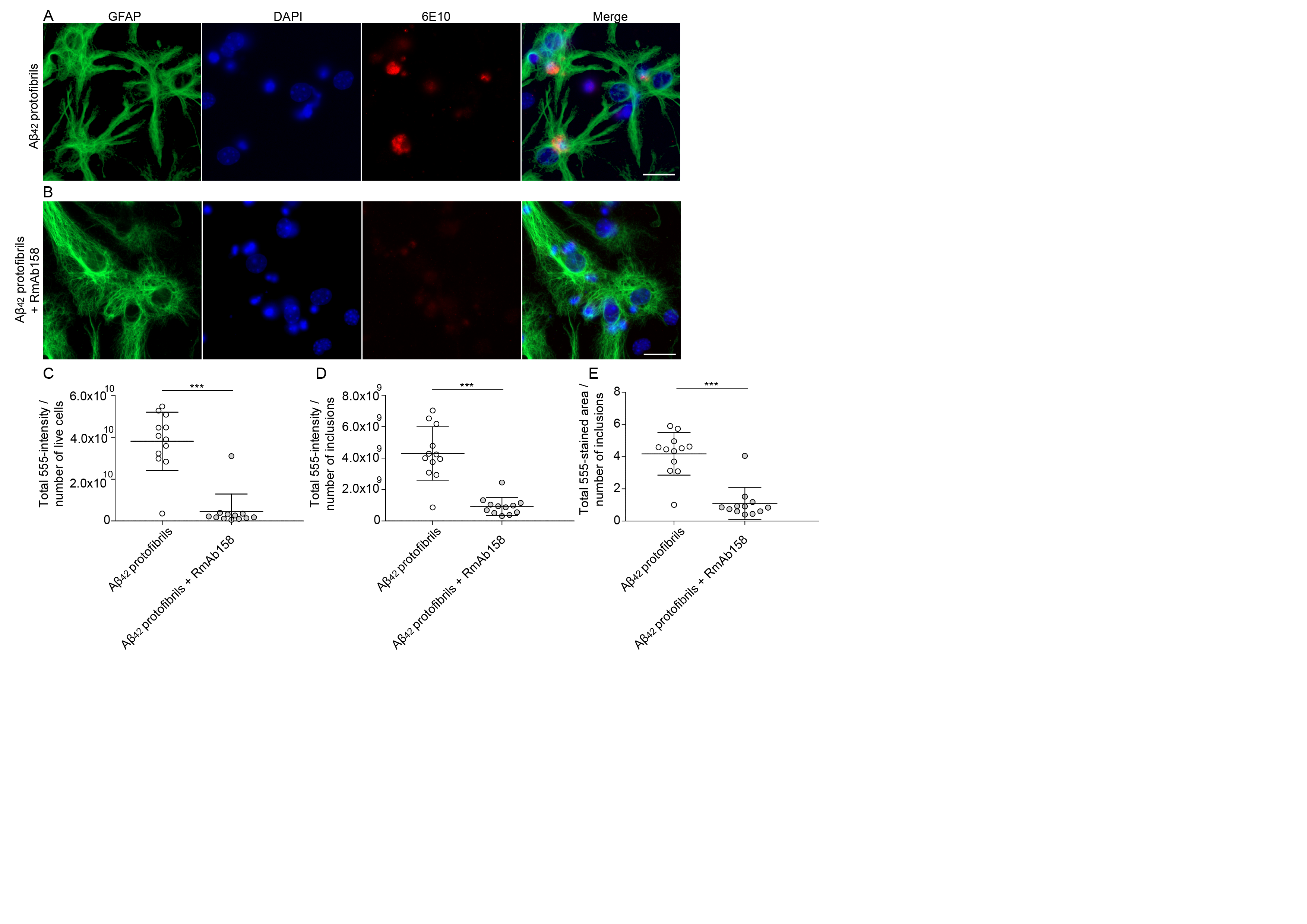

Supplement: Supplementary file 6 — RmAb158 reduces Aβ inclusions in astrocytes. Co-cultures were exposed to Aβ42 protofibrils (A) or Aβ42 protofibrils together with RmAb158 (IgG2c) (B). Measurements of the total 555-intensity per number of live cells (C) and number of inclusions (D), and the total 555-area per number of inclusions (E) confirmed that RmAb158 reduces Aβ inclusions in astrocytes. GFAP (green), DAPI (blue), Aβ (red). Scale bar: 20 μm. The experiments were performed in triplicates with independent cell cultures and 10 images/experiment were analyzed using Mann-Whitney U-test (***P < 0.001). (TIFF 4151 kb) [file 12974_2018_1134_MOESM6_ESM.tif]

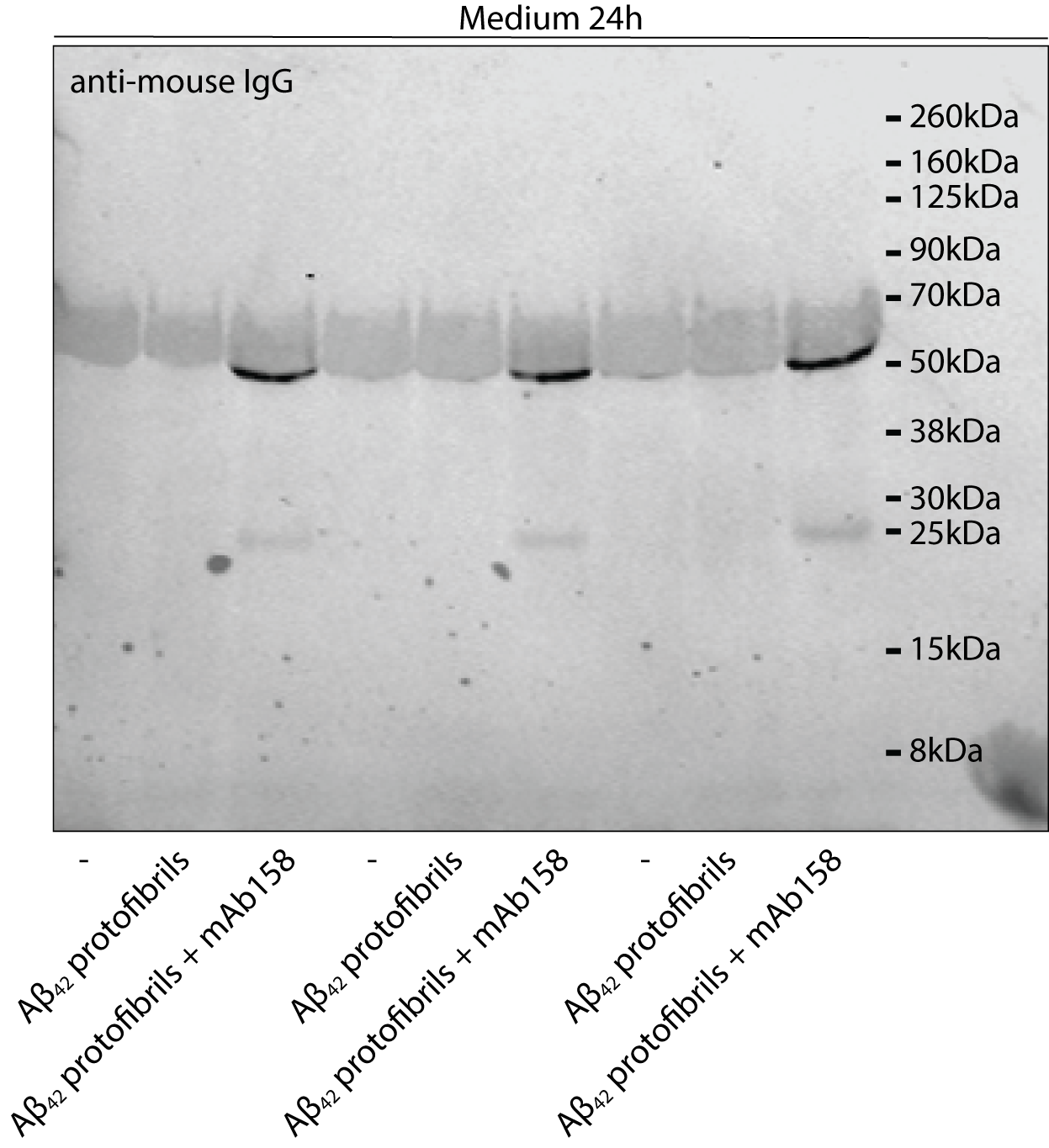

Supplement: Supplementary file 7 — The heavy and light chain of the mAb158 antibody can be detected in the media. Reprobing the filter in Fig. 5 with a secondary anti-mouse IgG antibody, showed that both the heavy and light chain of the mAb158 antibody can be detected in the media of antibody-treated cultures. (TIFF 2941 kb) [file 12974_2018_1134_MOESM7_ESM.tif]
